# The Role of Growth Factors in the Repair of Motor Injury

**DOI:** 10.3389/fphar.2022.898152

**Published:** 2022-05-19

**Authors:** Qiaoyin Tan, Jiayu Li, Yanmin Yin, Weide Shao

**Affiliations:** ^1^ College of Teacher Education, Zhejiang Normal University, Jinhua, China; ^2^ College of Physical Education and Health Sciences, Zhejiang Normal University, Jinhua, China

**Keywords:** growth factors, motor injury, repair of motor injury, post-injury rehabilitation, platelet-rich plasma

## Introduction

Growth factors are a group of bioactive peptides secreted by the body. Growth factors stimulate cell growth and exert other biological effects by binding to specific, highly affinity cell membrane receptors. There are various growth factors, such as platelet-like growth factors (platelet-derived growth factor, PDGF, Osteosarcoma-derived growth factor ODGF), epidermal growth factors (epidermal growth factor, EGF, transforming growth factor, TGFα, and TGFβ), fibroblast growth factors (αFGF, βFGF), insulin-like growth factors (IGF-I, IGF-II), nerve growth factor (NGF), interleukin-like growth factor (IL-1, IL-3, etc.), erythropoietin (EPO), colony stimulating factor (CSF), etc. Growth factors play irreplaceable roles in individual growth, development and aging, including accelerating metabolism, inhibiting inflammatory response, and repairing damaged tissues and organs.

The human motor system is mainly composed of nerves, muscles, bones, joints, and other organs and tissues, which is the basis of maintaining normal motor function. With the development of the society and the accumulation of life and work pressure, chronic diseases such as diabetes, cardiovascular and cerebrovascular diseases, and cancer are like a layer of haze hanging over the top of the head of the public. Proper exercise, as a brilliant “prescription”, plays an incomparable role in the treatment of chronic diseases in traditional Chinese medicine. The health of various sports tissues and organs is the prerequisite for the implementation of this prescription (Cacciata et al., 2019; Kirsch Micheletti et al., 2019). As an athlete, healthy sports function is an important guarantee for basic training and competition. Injuries in sports are common to both athletes and the public. For some severe athletic injuries, surgical treatment is considered the most effective way to restore motor function. However, the surgical trauma, long postoperative recovery time and functional limitation caused by scar tissue proliferation are all defects brought by surgery. In recent years, growth factors have been applied by scientists as a special treatment after exercise injury. The therapeutic effects and potentials demonstrated from *in vivo* animal tests to the current clinical applications are exciting. As a treatment with small trauma, mild side effects and easy operation, it has gradually replaced the traditional treatment in the treatment of part of the motor injury. Growth factors work admirably as a new treatment. At the same time, the difficulties and challenges in the future development of growth factors as a treatment for motor injury deserve attention.

## Function

### Muscle Injury

It is generally believed that the maintenance of skeletal muscle quality throughout life is the key to athletic health. Under normal physiological conditions, insulin-like growth factor -1 (IGF-1) can work together with androgens and mechanical muscle sensitivity to increase muscle mass (Gharahdaghi et al., 2020). During exercise, the relatively shallow positions of many muscles in the body make them more susceptible to acute injury from exposure to contusion, lacerations (Tidball, 2011). In addition, incorrect posture can also cause chronic muscle strain. The pathophysiological basis of muscle injury is mainly the direct mechanical damage to myofibrils caused by centrifugal contraction force and the destruction of muscle cell membrane (Tidball, 2011). There is evidence that a variety of growth factors play an important role in the repair of muscle injury (As shown in Table 1). Muscle satellite cells (MSCs), as the key participants in skeletal muscle regeneration, have the ability to secrete IGF-1 (Lee et al., 2018). IGF-1 secretion by MSCs is significantly increased after skeletal muscle injury. IGF-1 promotes the mitosis of myoblasts by mediating the mitogen-activated protein kinase (MAPK/ERK1/2) pathway and PI3K/Akt pathway, thereby repairing the damaged muscle cells in muscle fibers and then regenerating them (Ahmad et al., 2020). Like IGF-1, IGF-2 is necessary for muscle differentiation and development and functions in an autocrine manner with the same mechanism (Ahmad et al., 2020). Notably, the number of MSCs in each individual was different. William Zofkie et al. found that fibroblast growth factor 6 (FGF6) was necessary to regulate the number of MSCs in the postnatal period to establish a quiescent adult muscle stem cell bank (Zofkie et al., 2021). Another type of growth factor also plays an important role in muscle cell mitosis. Recently, an *in vitro* cytological study found that the supplementation of human epidermal growth factor (hEGF) in primary human myogenic cell cultures can promote the structural and functional characteristics of tissue-engineered skeletal muscle and enhance the proliferation and differentiation of skeletal muscle cells *in vitro* (Wroblewski et al., 2021). This latest research is of interest. HEGF cannot be obtained by autocrine of muscle cells, and biotechnology-dominated synthesis of hEGF reagents is expected to bring new treatment options for the repair of muscle injury. However, the specific mechanism of human epidermal growth factor (hEGF) acting on muscle cells needs further investigation. After the injury of myofibrils and muscle cells caused by centrifugal contractions, the damaged muscle recovers through different processes, including degeneration, inflammation, regeneration, and fibrosis (Quintero et al., 2009; Wong et al., 2015). TGF-β is a cytokine that participates in muscle recovery and repair. TGF-β can inhibit muscle regeneration, regulate extracellular matrix remodeling and promote fibrosis by regulating skeletal muscle inflammation. While some studies have shown that inhibition of TGF-β after muscle injury promotes muscle regeneration and recovery, others have pointed out that inhibition of TGF-β actually decreases muscle strength as it leads to incomplete muscle regeneration (Kim and Lee, 2017). Exercise injury may lead to degenerative atrophy of skeletal muscle. A study has found that hepatocyte growth factor (HGF) may regulate the transition of macrophages to M2 phenotype and promote the regeneration of skeletal muscle in mice (Choi et al., 2019). Autologous platelet-rich plasma (PRP) injection has been studied for a variety of applications, including as an adjunct therapy for muscle injury. Platelets release growth factors, including FGF-2, TGF-β1, PDGF, and IGF-1, and when platelets are highly concentrated by centrifugation, the resulting PRP solution is considered to improve tissue healing.

**TABLE 1:** The role of growth factors in the repair of motor injuries.

Organization	Growth factor and preparations	Function	Mechanism	Ref
Muscle	Insulin-like growth factor -1 (IGF-1)	Repair muscle damage and increase muscle quality	Activation of mitogen-activated protein kinase (MAPK/ERK1/2) pathway and PI3K/Akt pathway; Regulating the number of MSCs; Directly promote that proliferation and differentiation of muscle cells; Regulation of inflammatory response	Kim and Lee, (2017), Choi et al., (2019), Ahmad et al., (2020), Wroblewski et al., (2021), Zofkie et al., (2021)
	Insulin-like growth factor IGF-2			
	Fibroblast growth factor 6 (FGF6)			
	Human epidermal growth factor (hEGF)			
	Transforming growth factor -*β* (TGF-β)			
	Hepatocyte growth factor (HGF)			
	Autologous platelet rich plasma (PRP)			
Bones, joints	Fibroblast growth factor (FGF)	Repair damaged bones and protect bone homeostasis; Promote that regeneration of the joint synovial membrane; Repair damaged ligaments	Assisting the recruitment and migration of mesenchymal cells, the coagulation of progenitor cells, and the differentiation and maturation of chondrocytes; Promote the migration of bone progenitor cells and induce proliferation, differentiation and matrix formation; Induce osteoblast proliferation and differentiation	Frei et al., (2008), Kon et al., (2010), Liedert et al., (2014), Seijas et al., (2014), Zubair et al., (2018), Xu et al., (2022)
	Bone morphogenetic protein (BMP)			
	Insulin-like growth factor (IGF)			
	Platelet derived growth factor (PDGF)			
	Transforming growth factor -β(TGF-β)			
	Vascular endothelial growth factor (VEGF)			
	Autologous platelet rich plasma (PRP)			
	Growth factor midkine (Mdk)			
	Myelogenous growth factor (MYDGF)			
Nerve	Nerve growth factor (NGF)	Repair damaged nerve and neuromuscular junction	Regulating the expression of nerve factors at the injury site; Promoting mitosis and pluripotency of nerve cells; Reducing the death of nerve cells; Regulate autophagy to promote endothelial cell repair	Golzadeh and Mohammadi, (2016), Ko et al., (2019), Lu et al., (2020), Zhu et al., (2020), Ye et al., (2021), Yokozeki et al., (2021)
	TGF-β			
	Platelet derived growth factor B (PDGF-B)			
	Acidic fibroblast growth factor (aFGF)			
	Fibroblast growth factor 21 (FGF21)			
	Platelet derived growth factor (PDGF)			
	Vascular endothelial growth factor -A (Vegf-A)			

### Bone Damage

In the skeletal system, growth factors promote the production of large numbers of osteoblasts and inhibit osteoclasts, thereby ensuring the normal development of the skeletal system. Moreover, the role of growth factors in bone repair is widely recognized (see Table 1). Growth factors that act on bones, including mainly bone morphogenetic protein (BMP), fibroblast growth factor (FGF), insulin-like growth factor (IGF), platelet-derived growth factor (PDGF), transforming growth factor beta (TGF-β), and vascular endothelial growth factor (VEGF), are usually stored in the extracellular matrix (ECM), but are actively released by ECM, cells and platelets after injury^.^ (Devescovi et al., 2008). FGF is involved in the earliest stage-segment cartilage formation in bone development and is involved in assisting with the recruitment and migration of mesenchymal cells, the coagulation of progenitor cells, the differentiation and maturation of chondrocytes, and the formation of cartilage and bone during endochondral ossification (Goldring et al., 2006). BMP, PDGF, and IGF can promote the migration of bone progenitor cells and induce proliferation, differentiation and matrix formation (Devescovi et al., 2008). VEGF can promote cartilage to bone transformation, and can induce osteoblast proliferation and differentiation (Devescovi et al., 2008). TGF-β seems to have all the functions mentioned above during the process of bone repair. A Liedert et al. found that The growth factor midkine (Mdk) plays a key role in bone remodeling, and it is expressed in the processes of bone formation and fracture repair (Liedert et al., 2014). A recent *in vivo* animal study reported that myelogenous growth factor (MYDGF) protects bone mass by inhibiting osteoclastogenesis and promoting osteoblast differentiation, and is a positive modulator of bone homeostasis (Xu et al., 2022). Similarly, growth factors play an important role in the repair of joint injuries. A study reported by Ling Yu pointed out that BMP9 can stimulate the regeneration of joint synovial membrane and achieve the reconstruction of joint function. Autologous platelet-rich plasma (PRP) is the most widely used agent for the treatment of joint injuries and its effects are well recognized (Zubair et al., 2018). R Frei et al. demonstrated a favorable therapeutic effect of PRGF-rich plasma for the treatment of ankle ligament complex injury in a retrospective clinical trial. It can be used as a replacement for traditional surgery or as an adjunct to accelerate and improve the healing of traumatic lesions and postoperative conditions (Frei et al., 2008). Consistently, Roberto Seijas et al. by treating a patient with a partial anterior cruciate ligament tear with growth factor-rich intraligamentous plasma, they found that a professional soccer player’s athletic ability could be restored to the level prior to the injury with the retention of an intact cingulate band (Seijas et al., 2014). In addition, PDGF also has a good effect in the treatment of degenerative diseases in the elderly. One study has confirmed that intra-articular injection of PRP is safe and has the potential to reduce the degree of joint degeneration, improving pain, and knee function and quality of life in young patients (Kon et al., 2010). Current sources for delivery of the GF mixture to bone repair sites are platelet gel and demineralized bone matrix. However, GF levels in these formulations were affected by donor-to-donor variability and formulation differences. Autologous GF generated by patients themselves during bone repair may interfere with the prosthetic device and even cause the implant to become loose due to tissue reaction around the prosthesis. In conclusion, GF is a key component of functional bone regeneration: Screening of basic research results and controlled clinical trials are accelerating the development of GF in orthopedic surgery.

### Nerve Injury

In the nervous system, growth factors have the function of promoting the generation of brain nerve cells and dendrites, and enhancing the electrochemical signal transduction between the nerves and muscles. After motor injury, especially in the traumatic injury caused by violence, the nerve and neuromuscular junction at the injury site will be damaged, leading to the related complications. Some studies have found that some growth factors play an important role in the repair of nerve injury (see Table 1). Nerve growth factor (NGF) is best known for its remarkable effects on the repair of nerve cells (Jafari et al., 2019). Although TGF-β cannot directly participate in the repair after nerve injury, it can play a positive role by regulating the expression of nerve factors at the injury site (Yokozeki et al., 2021). The results of an animal model study conducted by Atefeh Golzadeh et al. showed that local administration of platelet-derived growth factor B (PDGF-B) had a beneficial effect on the regeneration and functional recovery of peripheral nerves, but the specific biological mechanism was still unclear (Golzadeh and Mohammadi, 2016). Spinal cord injury is the most serious injury to motor-related nerves. The repair of damaged nerves by acidic fibroblast growth factor (aFGF) is due to its ability to promote mitosis and pluripotency of nerve cells, thus playing a role in the repair of nerves (Ko et al., 2019). Fibroblast growth factor 21 (FGF21) can also play a role in repairing nerves, mainly by reducing the death of nerve cells and promoting the healing of nerve injury (Zhu et al., 2020). A recent study has shown that exogenous platelet-derived growth factor (PDGF) can improve the recovery of neurovascular units after spinal cord injury by regulating autophagy to promote endothelial cell repair (Ye et al., 2021). There are also relevant literature reports on the role of growth factors in the repair of neuromuscular junction injury. Chuien-yilu et al. found through *in vivo* animal experiments that macrophage-derived vascular endothelial growth factor -A (Vegf-A) is a key component of recovery after neuromuscular junction injury, revealing a new therapeutic goal for repair after motor injury (Lu et al., 2020). Although some growth factors have not yet been studied in humans for the repair of neurological function, a positive effect on this aspect is expected.

## Discussion

Growth factors have shown unique clinical advantages in the treatment of motor injury. Currently, platelet-rich plasma (PRP) is the most widely used growth factor biologics and is a natural concentrate of growth factors derived from autologous blood. It has good curative effect in the treatment of various osteoarthritis, long-term chronic sports injury, pain caused by degenerative changes, and postoperative rehabilitation. Synergy between growth factors is feasible, and in addition to PRP, BMP-2 in combination with VEGF has been shown to play a positive role in the repair of muscle and bone injuries (Subbiah et al., 2020). Growth factors not only play an important role in the repair of muscle, bone and nerve injuries, but also play a potential role in the relief of post-injury pain. Previous studies have demonstrated the potential role of insulin-like growth factor (IGF) in the treatment of myofascial pain syndrome. Compared with traditional therapy, growth factor preparation therapy has the characteristics of less trauma, rapid recovery and good patient compliance. Although growth factors and their preparations are increasingly widely used in the treatment of motor injury, the specific mechanism is still not very clear. In particular, the specific role of each growth factor in motor injury should be elucidated. The accurate mechanism is to achieve the purpose of accurate treatment according to the injury situation in future treatment. Individual derived growth factors are limited and it is necessary to prepare growth factors and related formulations by other means. With the development of genetic engineering and biotechnology engineering, the demand for growth factors and their preparations will be met in the future. It is worth worrying that as a bioengineering preparation, its possible safety and usage standardization should be considered. In general, the effect of growth factors on exercise health is irreplaceable, and its prospect is bright. However, the understanding of the relationship between them is vague.
